# Impaired Fibrinolysis Predicts Adverse Outcome in Acute Coronary Syndrome Patients with Diabetes: A PLATO Sub-Study

**DOI:** 10.1055/s-0039-1701011

**Published:** 2020-01-23

**Authors:** Wael Sumaya, Lars Wallentin, Stefan K. James, Agneta Siegbahn, Katja Gabrysch, Anders Himmelmann, Ramzi A. Ajjan, Robert F. Storey

**Affiliations:** 1 Department of Infection, Immunity and Cardiovascular Disease, University of Sheffield, Sheffield, United Kingdom; 2 Department of Medical Sciences, Cardiology, Uppsala University, Uppsala, Sweden; 3 Uppsala Clinical Research Center, Uppsala University, Uppsala, Sweden; 4 Department of Medical Sciences, Clinical Chemistry, Uppsala University, Uppsala, Sweden; 5 AstraZeneca Research and Development, Gothenburg, Sweden; 6 Leeds Institute of Cardiovascular and Metabolic Medicine, University of Leeds, Leeds, United Kingdom

**Keywords:** acute coronary syndrome, diabetes, fibrinolysis

## Abstract

Hypofibrinolysis is a key abnormality in diabetes but the role of impaired clot lysis in predicting vascular events and mortality in this population is yet to be determined. We aimed to investigate the relationship between fibrin clot properties and clinical outcomes in patients with diabetes and recent acute coronary syndrome (ACS). Plasma samples were collected at hospital discharge from 974 ACS patients with diabetes randomised to clopidogrel or ticagrelor in the PLATO trial. A validated turbidimetric assay was employed to study fibrin clot lysis and maximum turbidity. One-year rates of cardiovascular (CV) death, spontaneous myocardial infarction (MI) and PLATO-defined major bleeding events were assessed after sample collection. Hazard ratios (HRs) were determined using Cox proportional analysis. After adjusting for CV risk factors, each 50% increase in lysis time was associated with increased risk of CV death/MI (HR 1.21; 95% confidence interval [CI] 1.02–1.44; *p* = 0.026) and CV death alone (HR 1.38; 1.08–1.76; *p* = 0.01). Similarly, each 50% increase in maximum turbidity was associated with increased risk of CV death/MI (HR 1.25; 1.02–1.53; *p* = 0.031) and CV death alone (HR 1.49; 1.08–2.04; *p* = 0.014). The relationship between lysis time and the combined outcome of CV death and MI remained significant after adjusting for multiple prognostic vascular biomarkers ( *p* = 0.034). Neither lysis time nor maximum turbidity was associated with major bleeding events. Impaired fibrin clot lysis predicts 1-year CV death and MI in diabetes patients following ACS.

**Clinical Trial Registration** URL: http://www.clinicaltrials.gov . Unique identifier NCT00391872.

## Introduction

Up to 30% of patients presenting with acute coronary syndrome (ACS) suffer from diabetes mellitus. 1 2 These individuals have worse vascular outcomes despite contemporary therapies. 1 3 4 A ‘pro-thrombotic’ state, characterised by adverse fibrin clot properties and increased platelet reactivity, has been repeatedly described in patients with diabetes. 5 6 7 Despite the altered thrombotic milieu in this condition, long-term preventative anti-thrombotic treatment post-ACS remains similar compared with individuals without diabetes. 8 9 Offering more intensive therapies may be one approach to improve outcomes, but this can be challenging in diabetes given the heterogeneity of this condition, which is characterised by variable risks of thrombosis and bleeding. 10

Potent P2Y _12_ inhibitors (ticagrelor/prasugrel) post-ACS improved outcomes in patients with diabetes without an apparent penalty of increased major bleeding compared with clopidogrel. 4 11 Targeting the protein arm of coagulation with low-dose anti-factor Xa therapy, in addition to clopidogrel-based dual anti-platelet therapy, was shown to reduce cardiovascular (CV) events and mortality in ACS patients, regardless of diabetes status. 12 However, the observed increase in bleeding and the guideline-recommended use of dual anti-platelet therapy with ticagrelor or prasugrel, rather than clopidogrel, has limited widespread adoption of this approach.

Clinical characteristics, such as the extent of coronary artery disease, history of recurrent events or renal impairment, and elevated CV biomarkers could help guide intensity of treatment, 13 but functional biomarkers that address thrombosis risk are lacking. Identification of such biomarkers could potentially make it possible to implement tailored anti-thrombotic therapy in this population, helping to maximise benefits and minimise risks.

Cross-sectional studies have repeatedly shown a relationship between coronary artery disease and dense fibrin networks that are resistant to lysis. 14 15 16 These associations were documented in individuals with and without diabetes, although the latter group was generally found to have a more thrombotic clot phenotype. 17 18 19 We have recently demonstrated that impaired fibrin clot lysis independently predicts CV death following ACS, 20 indicating that the fibrin network has clinical prognostic significance. Diabetes was also associated with impaired fibrin clot lysis but the magnitude of the association between prolonged fibrin clot lysis and adverse outcomes in diabetes patients was not assessed. 20 In this sub-analysis, we aimed to assess the association between fibrin network properties and adverse clinical outcome in ACS patients with diabetes.

## Methods

### Study Population and Patient Samples

The PLATelet inhibition and patient Outcomes (PLATO) trial was an international multi-centre, double-blind, randomised controlled trial of ticagrelor compared with clopidogrel in 18,624 moderate-to-high-risk ACS patients. 21 22 Study design and results have been previously reported. 21 22 Briefly, patients admitted with ACS were recruited within 24 hours of symptom onset and randomised to either clopidogrel or ticagrelor. Patients were followed up at 1 to 3, 6 to 9 and 12 months. The PLATO fibrin sub-study included 4,354 patients who donated blood at hospital discharge. 20 This is a sub-group analysis involving all 974 patients with diabetes. Citrated plasma was derived and stored at –80°C at Uppsala Clinical Research Centre (Uppsala, Sweden) prior to transfer to the University of Sheffield (Sheffield, United Kingdom) for fibrin clot analysis.

### Fibrin Clot Assessment

This was performed using a turbidimetric assay as previously described. 20 Briefly, plasma mixed with tissue plasminogen activator (tPA) (83 ng/mL) was re-calcified (CaCl _2_ 7.5 mM) and clotting was initiated with thrombin (0.03 U/mL). Fibrin clot maximum turbidity (a measure of fibrin clot density) and lysis time were determined using a Multiskan FC (Thermo scientific) plate reader in all 974 plasma samples taken at hospital discharge and 820 plasma samples taken at 1 month. All laboratory analysis was performed blinded to clinical outcomes, treatment allocation and other biomarker levels.

### Statistical Methods

Biomarker levels were natural log transformed before analysis. Continuous data are presented as medians and interquartile ranges and compared using Kruskal–Wallis tests, Wilcoxon tests or multivariable linear regression models, as appropriate. Categorical data are presented as numbers and percentages and compared using chi-square tests. The primary outcome of interest was the composite of CV death and spontaneous myocardial infarction (MI). Secondary outcomes were CV death alone, MI alone, PLATO-defined major bleeding and all-cause mortality. Cox-proportional hazards models were used to estimate hazard ratios (HRs) and 95% confidence intervals (CIs). HRs are expressed per 50% fibrin variable level increase. We adjusted for clinical risk factors (model 1) and for known prognostic biomarkers. Model 1 included all clinical risk factors, including randomised treatment, age, gender, body mass index (BMI), smoking history, hypertension, dyslipidaemia, chronic kidney disease (CKD), ST-elevation ACS and previous MI, congestive heart failure, revascularisation, ischaemic stroke or peripheral artery disease. Prognostic biomarkers were added sequentially to adjustment model 1. Model 2 included adjustment for model 1, white cell count and C-reactive protein (CRP). Model 3 included adjustment for model 1 clinical risk except for CKD, white cell count, CRP and cystatin C. Model 4 included adjustment for model 3 risk factors, troponin T and N-terminal pro B-type natriuretic peptide (NT-proBNP). Model 5 included all model 4 risk factors and growth differentiation factor-15. Restricted cubic splines were used to visually assess the relationship between fibrin clot properties and clinical outcomes. Interactions between prognostic value of fibrin clot parameters and each of randomised treatment, treatment strategy and treatment with low-molecular-weight heparin (LMWH) (within 2 days of sampling) were assessed using restricted cubic splines. Mean ± standard deviation fibrin clot properties at hospital discharge and 1 month were compared using Wilcoxon signed-rank test. *p* -Values < 0.05 from two-tailed tests were considered statistically significant. *p* -Values were not adjusted for multiple testing. All statistical analyses were performed at Uppsala Clinical Research Centre using R statistics software (Version 3.3.2; R Foundation for Statistical Computing, Vienna, Austria).

## Results

### Diabetes, Associated Conditions and Biomarkers

Table 1 outlines the difference between patients with and without diabetes. Diabetes showed associations with CV risk factors, including increased age, increased BMI, hypertension, hyperlipidaemia, CKD and peripheral artery disease. However, smoking was less prevalent in diabetes patients. Higher proportions presented with non-ST-elevation ACS, were females and had previous MI, stroke, congestive heart failure or revascularisation compared with patients without diabetes.

**Table 1:** Clinical characteristics in diabetes and non-diabetes patients

Variable	Diabetes *n* = 974	No diabetes *n* = 3,380	*p* -Value
Demographics and risk factors			
Age (y)	64 (56–72)	61 (53–70)	< 0.001
Females	342 (35.1%)	931 (27.5%)	< 0.001
Body mass index (kg/m ^2^ )	29 (26–33)	27 (25–30)	< 0.001
Current smoker	232 (23.8%)	1363 (40.3%)	< 0.001
Hypertension	806 (82.8%)	2059 (60.9%)	< 0.001
Hyperlipidaemia	533(54.7%)	1307(38.7%)	< 0.001
Previous MI	261 (26.8%)	585 (17.3%)	< 0.001
Congestive heart failure	86 (8.8%)	163 (4.8%)	< 0.001
Previous PCI	153 (15.7%)	375 (11.1%)	< 0.001
Previous CABG	78 (8.0%)	143 (4.2%)	< 0.001
Previous stroke	48 (4.9%)	103 (3.0%)	0.005
Peripheral artery disease	82 (8.4%)	191 (5.7%)	0.002
CKD	62 (6.4%)	85 (2.5%)	< 0.001
Randomised treatment			
Ticagrelor	490 (50.3%)	1687 (49.9%)	0.83
Presentation			
STE-ACS	354 (36.3%)	1668 (49.3%)	< 0.001

Abbreviations: CABG, coronary artery bypass graft; CKD, chronic kidney disease; MI, myocardial infarction; PCI, percutaneous coronary intervention; STE-ACS, ST-elevation acute coronary syndrome.

Note: Values are medians (interquartile ranges [IQRs]) for continuous data and *n* (%) for categorical data. *p* -Values were calculated using Wilcox test for age and chi - square test for categorical variable.

After adjustment for risk factors and clinical characteristics (model 1), fibrin clot lysis time and maximum turbidity were significantly higher in patients with diabetes compared with those without. The majority of other prognostic and inflammatory biomarkers were significantly higher in patients with diabetes ( Table 2 ).

**Table 2:** Biomarker levels in diabetes and non-diabetes patients

Biomarker	Diabetes *n* = 974	No diabetes *n* = 3,380	Adjusted *p* -value
Lysis time (s)	732 (594–1,002)	684 (558–864)	< 0.001
Maximum turbidity (AU)	0.51 (0.39–0.64)	0.49 (0.38–0.62)	0.004
Troponin T (ng/L)	521 (105–1,535.5)	716 (123.5–2,264)	0.2
NT-proBNP (pmol/L)	621.5 (247–1,578)	562.5 (236–1,253)	< 0.001
Cystatin C (mg/L)	1.0 (0.8–1.2)	0.9 (0.8–1.1)	0.14
GDF-15	1,931 (1422–2,935)	1,469 (1,121–2,062)	< 0.001
CRP (mg/L)	15 (5.9–35)	14 (5.3–32)	< 0.001
IL-6	5.8 (3.3–11)	5.5 (2.9–10)	0.003
WCC	9.2 (7.6–11.5)	9.4 (7.4–11.7)	< 0.001
HbA1c	7.5 (6.6–8.9)	5.9 (5.6–6.2)	< 0.001

Abbreviations: AU, arbitrary unit; CRP, C-reactive protein; GDF-15, growth differentiation factor 15; HbA1C, glycated haemoglobin; IL, interleukin; NT-proBNP, N-terminal pro B-type natriuretic peptide; WCC, white cell count.

Note: *p* -Values were calculated using multivariable linear regression analysis with adjustment to all clinical characteristics (model 1). All biomarkers were measured at hospital discharge except for WCC and HbA1c which were measured at baseline.

### Fibrin Clot Properties, Clinical Characteristics and Biomarker Levels

The correlation between fibrin clot maximum turbidity and lysis time was weak ( *r* = 0.37, *p* < 0.001).

Tables 3 and 4 summarise clinical characteristics and biomarker levels across the four quartile groups of fibrin clot properties. BMI and the proportion of females increased with increasing quartile groups of lysis time. Similarly, the prevalence of CKD was highest in the highest lysis time quartile group. Levels of prognostic and inflammatory biomarkers significantly increased with increasing quartile groups of both lysis time and maximum turbidity.

**Table 3:** Clinical characteristics and biomarkers across quartile groups of lysis time in patients with diabetes

Variables	Lysis time (s) quartile group				*p* -Value
	Q1 (< 594) *n* = 251	Q2 (594–732) *n* = 239	Q3 (732–1,002) *n* = 243	Q4 (> 1,002) *n* = 241	
Demographics and medical history					
Age (y)	65 (58–72)	65 (57–72)	64 (56–72)	64 (55–71)	0.25
Female	67 (26.7%)	73 (30.5%)	84 (34.6%)	118 (49.0%)	< 0.001
BMI (kg/m ^2^ )	27.8 (25.5–31.8)	28.7 (26.4–32.5)	29.4 (26.4–32.4)	29.8 (26.3–33.9)	0.004
Hypertension	188 (74.9%)	208 (87.0%)	206 (84.8%)	204 (84.6%)	0.002
Previous MI	68 (27.1%)	61 (25.5%)	71 (29.2%)	61 (25.3%)	0.75
Previous stroke	7 (2.8%)	13 (5.4%)	13 (5.3%)	15 (6.2%)	0.31
PAD	14 (5.6%)	23 (9.6%)	23 (9.5%)	22 (9.1%)	0.31
CKD	8 (3.2%)	15 (6.3%)	15 (6.2%)	24 (10.0%)	0.02
Treatment strategy					
Invasive	159 (63.3%)	146 (61.1%)	164 (67.5%)	157 (65.1%)	0.51
Inpatient PCI	152 (60.6%)	140 (58.6%)	154 (63.4%)	146 (60.6%)	0.76
Inpatient CABG	8 (3.2%)	6 (2.5%)	10 (4.1%)	12 (5%)	0.5
Ticagrelor	118 (47%)	114 (47.7%)	130 (53.5%)	128 (53.1%)	0.32
Supine systolic BP a	140 (120–150)	140 (120–150)	140 (120–150)	140 (121–152)	0.64
Biomarkers					
Troponin T (ng/L)	326 (86–1,186)	371 (73–1,415)	604 (139–1,602)	703 (149–1,868)	0.01
NT-proBNP (pmol/L)	486 (238–1,052)	602 (222–1,358)	760 (307–2,118)	716 (279–2,197)	0.001
Cystatin C (mg/L)	0.92 (0.77–1.15)	0.94 (0.76–1.16)	0.94 (0.76–1.26)	1.04 (0.86–1.38)	< 0.001
GDF-15 (ng/L)	1,842 (1,435–2,651)	1,864 (1,414–2,741)	1,933 (1,392–2,891)	2,139 (1,479–3,689)	0.01
CRP (mg/L)	9 (4–21)	14 (5–27)	17 (8–52)	24 (11–58)	< 0.001
WCC (× 10 ^9^ /L)	8.5 (7.1–10.6)	9.4 (7.8–11.3)	9.3 (8.0–11.8)	9.9 (7.8–12.1)	< 0.001
Platelets (× 10 ^9^ /L)	218 (191–254)	235 (197–274)	235 (194–284)	251 (203–304)	< 0.001
HbA1c (%)	7.3 (6.5–8.6)	7.4 (6.4–9)	7.5 (6.6–9)	7.8 (6.8–9.1)	0.02
Glucose (mmol/L)	9.3 (7.2–12.4)	9.7 (7.3–13.8)	9.3 (7.5–13)	10.4 (7.8–13.2)	0.13
LDL (mmol/L)	2.7 (2.1–3.4)	2.8 (2.2–3.5)	3.0 (2.2–4.0)	3.0 (2.4–4.0)	0.008
HDL (mmol/L)	1.2 (1.0–1.4)	1.1 (0.9–1.4)	1.2 (1.0–1.4)	1.2 (1.0–1.3)	0.31
Pre-admission insulin treatment	56 (22.3%)	54 (22.6%)	55 (22.6%)	60 (24.9%)	0.9
Insulin treatment during admission	122 (48.6%)	115 (48.1%)	124 (51%)	139 (57.7%)	0.14

Abbreviations: BMI, body mass index; BP, blood pressure; CABG, coronary artery bypass graft; CKD, chronic kidney disease; CRP, C-reactive protein; GDF-15, growth differentiation factor-15; HbA1c, glycated haemoglobin; HDL, high-density lipoprotein; LDL, low-density lipoprotein; MI, myocardial infarction; NT-proBNP, N-terminal pro B-type natriuretic peptide; PAD, peripheral artery disease; PCI, percutaneous coronary intervention; WCC, white cell count.

Note: Values are medians (interquartile ranges [IQRs]) for continuous data and *n* (%) for categorical data. *p* -Values calculated using chialculat test for categorical variables and Kruskal–Wallis test for continuous variables. Biomarkers measures at hospital discharge except for WCC, platelets, HbA1c, glucose and cholesterol levels which were measured at baseline.

a Blood pressure measured at baseline.

**Table 4:** Clinical characteristics and biomarkers across quartile groups of maximum turbidity in patients with diabetes

Variables	Maximum turbidity (AU) quartile group				*p* -Value
	Q1 (< 0.39) *n* = 244	Q2 (0.39–0.51) *n* = 243	Q3 (0.51–0.64) *n* = 244	Q4 (> 0.64) *n* = 243	
Demographics and medical history					
Age (y)	64 (56–72)	65 (57–72)	63 (56–71)	64 (57–71)	0.66
Female	88 (36.1%)	95 (39.1%)	86 (35.2%)	73 (30.0%)	0.21
BMI (kg/m ^2^ )	28.4 (25.6–32.7)	29.1 (26.3–32.7)	29.1 (26.3–33.2)	29.1 (26.0–32.3)	0.71
Hypertension	203 (83.2%)	207 (85.2%)	195 (79.9%)	201 (82.7%)	0.49
Previous MI	74 (30.3%)	76 (31.3%)	62 (25.4%)	49 (20.2%)	0.02
Previous stroke	8 (3.3%)	14 (5.8%)	12 (4.9%)	14 (5.8%)	0.54
PAD	20 (8.2%)	24 (9.9%)	14 (5.7%)	24 (9.9%)	0.30
CKD	12 (4.9%)	14 (5.8%)	15 (6.1%)	21 (8.6%)	0.37
Treatment strategy					
Invasive	120 (49.2%)	156 (64.2%)	161 (66%)	189 (78%)	< 0.001
Inpatient PCI	114 (46.7%)	152 (62.6%)	153 (62.7%)	173 (71.2%)	< 0.001
Inpatient CABG	6 (2.5%)	4 (1.6%)	8 (3.3%)	18 (7.4%)	0.004
Ticagrelor	119 (48.8%)	106 (43.6%)	137 (56.1%)	128 (52.7%)	0.04
Supine systolic BP	140 (120–150)	140 (123–150)	140 (120–153)	140 (125–150)	0.95
Biomarkers					
Troponin T (ng/L)	148 (37–579)	337 (74–1,133)	645 (193–1,708)	1,223 (525–2,781)	< 0.001
NT-proBNP (pmol/L)	345 (171–797)	535 (217–1,130)	590 (299–1,565)	1,409 (552–2,934)	< 0.001
Cystatin C (mg/L)	0.93 (0.77–1.15)	0.96 (0.78–1.21)	0.94 (0.77–1.19)	1.0 (0.83–1.37)	0.02
GDF-15 (ng/L)	1,765 (1,323–2,527)	1,926 (1,441–2,766)	1,917 (1,412–2,892)	2,268 (1,576–3,759)	<,0.001
CRP (mg/L)	5 (3–13)	10 (5–18)	18 (10–32)	50 (26–100)	< 0.001
WBC (× 10 ^9^ /L)	8.1 (6.9–9.9)	8.8 (7.4–11.1)	9.4 (7.8–11.1)	10.7 (9–13.5)	< 0.001
Platelets (× 10 ^9^ /L)	228 (201–263)	227 (192–273)	233 (198–276)	240 (194–285)	0.12
HbA1c (%)	7.3 (6.4–8.7)	7.6 (6.7–9.4)	7.6 (6.6–9)	7.5 (6.7–8.7)	0.27
Glucose (mmol/L)	9.0 (6.7–12.1)	9.5 (7.3–12.4)	9.7 (7.4–13)	10.5 (8–13.8)	0.004
LDL (mmol/L)	2.9 (2.1–3.5)	2.8 (2.2–3.6)	2.9 (2.3–3.9)	3.0 (2.3–3.7)	0.38
HDL (mmol/L)	1.1 (1–1.4)	1.2 (1–1.4)	1.2 (1–1.4)	1.2 (1–1.4)	0.41
Pre-admission insulin treatment	53 (21.7%)	57 (23.5%)	60 (24.6%)	55 (22.6%)	0.9
Insulin treatment during admission	114 (46.7%)	119 (49%)	129 (52.9%)	138 (56.8%)	0.12

Abbreviations: AU, arbitrary unit; BMI, body mass index; BP, blood pressure; CABG, coronary artery bypass graft; CKD, chronic kidney disease; CRP, C-reactive protein; GDF-15, growth differentiation factor-15; HbA1c, glycated haemoglobin; HDL, high-density lipoprotein; LDL, low-density lipoprotein; NT-proBNP, N-terminal pro B-type natriuretic peptide; MI, myocardial infarction; PAD, peripheral artery disease; PCI, percutaneous coronary intervention; WCC, white cell count.

Note: Values are medians (interquartile ranges [IQRs]) for continuous data and *n* (%) for categorical data. *p* -Values calculated using calculated test for categorical variables and Kruskal–Wallis test for continuous variables.

Platelet count and low-density lipoprotein (LDL) cholesterol levels increased with increasing fibrin clot lysis time but showed no relationship to maximum turbidity. There was no significant difference in the proportion of patients receiving ticagrelor across the four lysis time quartile groups.

The correlation between lysis time and the inflammatory biomarker CRP was weak ( *r* = 0.29, *p* < 0.001). In contrast, the relationship between fibrin clot maximum turbidity and CRP appeared more linear ( *r* = 0.63, *p* < 0.001). Both lysis time ( *r* = 0.09, *p* = 0.005) and maximum turbidity ( *r* = 0.41, *p* < 0.001) were significantly, though weakly, correlated with troponin T.

Glycated haemoglobin (HbA1c) also significantly increased with increasing lysis time ( Table 2 ) but no association with maximum turbidity was found ( Table 4 ). Insulin treatment did not show associations with fibrin clot properties.

### Fibrin Clot Properties and Clinical Outcomes

During follow-up, 48 patients (4.9%) had CV death, 72 (7.4%) had MI, 67 (2.9%) had major bleeding and 21 (2.2%) had non-coronary artery bypass graft-related major bleeding.

**Fig. 1:**
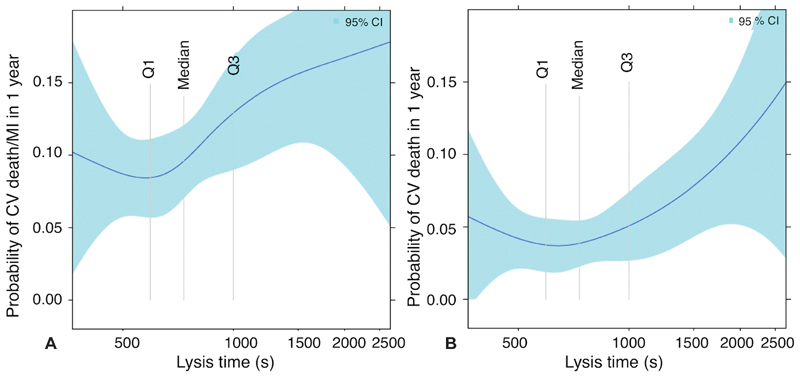
Relationship between fibrin clot lysis time and 1-year clinical outcomes in patients with diabetes mellitus, 1-year rates of cardiovascular (CV) death and spontaneous myocardial infarction (MI) ( **A** ) and CV death alone ( **B** ) in relation to lysis time transformed using restricted cubic splines. Shaded areas represent 95% confidence intervals. Vertical lines indicate quartiles.

The probability of the combined outcome of CV death and MI was higher with increasing values of lysis time ( Fig. 1A ). This was driven primarily by increased risk of CV death with increasing lysis time ( Fig. 1B ). There was no clear relationship between lysis time and major bleeding events.

**Fig. 2:**
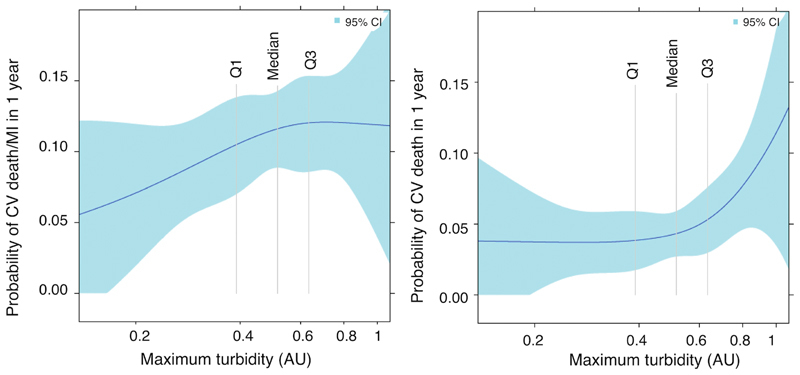
Relationship between fibrin clot maximum turbidity and 1-year clinical outcomes in patients with diabetes mellitus, 1-year rates of cardiovascular (CV) death and spontaneous myocardial infarction (MI) ( **A** ) and CV death alone ( **B** ) in relation to maximum turbidity (AU, arbitrary units) transformed using restricted cubic splines. Shaded areas represent 95% confidence intervals. Vertical lines indicate quartiles.

Similarly, the probability of the combined outcome of CV death and MI was higher with increasing maximum turbidity ( Fig. 2A ). The highest quartile group, in particular, appeared to be associated with greatest risk of CV death ( Fig. 2B ). In contrast, the probability of major bleeding seemed to increase with decreasing maximum turbidity.

**Fig. 3:**
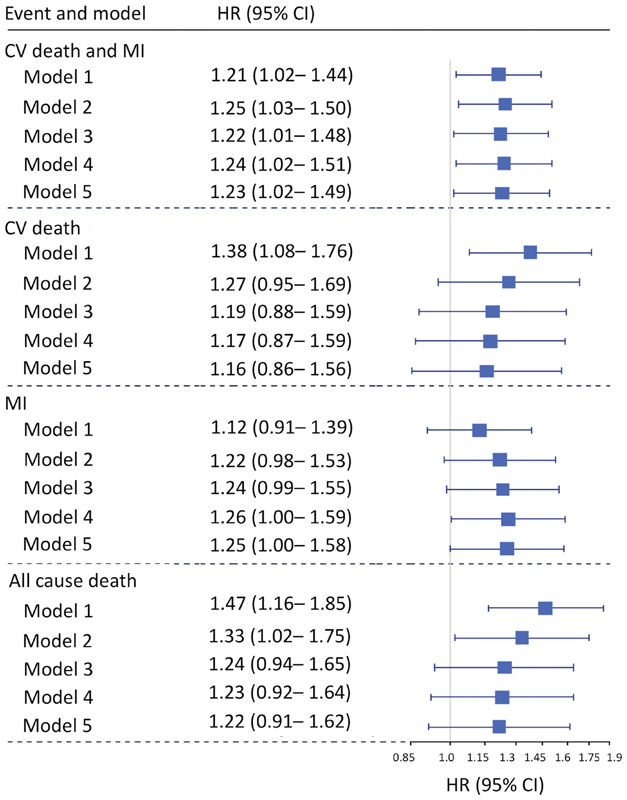
Forest plot for the associations between fibrin clot lysis time and clinical outcomes following acute coronary syndrome (ACS) in patients with diabetes. Squares represent hazard ratio (HR) estimates. Horizontal lines represent 95% confidence intervals. Number of patients, 971 for model 1 and 853 for subsequent models. Model 1: Clinical characteristics including randomised treatment, age, gender, body mass index (BMI), smoking history, hypertension, dyslipidaemia, chronic kidney disease (CKD), ST-elevation ACS and previous MI, congestive heart failure, revascularisation, ischaemic stroke or peripheral artery disease; Model 2: Clinical characteristics as per model 1 + C-reactive protein (CRP) + white cell count (WCC); Model 3: All characteristics and biomarkers as per model 2 (except CKD) + cystatin C; Model 4: All characteristics and biomarkers as per model 3 + troponin + N-terminal pro B-type natriuretic peptide (NT-proBNP); Model 5: All characteristics and biomarkers as per model 4 + growth differentiation factor 15 (GDF-15).

After adjustment for clinical characteristics and CV disease risk factors (model 1), each 50% increase in lysis time was associated with increased risk of CV death/MI (HR 1.21; 95% CI 1.02–1.44) and CV death alone (HR 1.38; 95% CI 1.08–1.76). Similarly, every 50% increase in lysis time was associated with increased risk of all-cause mortality after adjustment for clinical risk factors in model 1 (HR 1.47; 95% CI 1.16–1.85) and this remained significant after adjustment for inflammatory biomarkers ( Fig. 3 ). After adjustment for prognostic biomarkers as well as clinical risk factors (model 5), the relationship between lysis time and the combined outcome of CV death and MI remained significant ( *p* = 0.034).

**Fig. 4:**
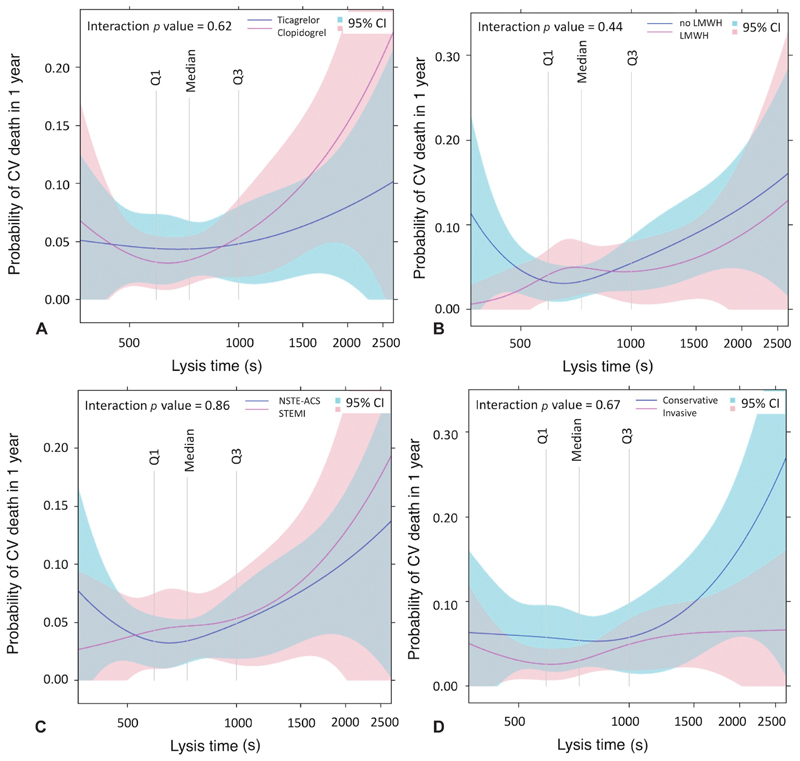
Relationship between fibrin clot lysis time and cardiovascular death according to randomised treatment ( **A** ), low-molecular-weight heparin treatment ( **B** ), presentation ( **C** ) and treatment strategy ( **D** ). One-year rates of cardiovascular death in relation to lysis time, transformed using restricted cubic splines. Shaded areas represent 95% confidence intervals, vertical lines indicate quartiles. LMWH, low-molecular-weight heparin; NSTE-ACS, non-ST-elevation acute coronary syndrome; STEMI, ST-elevation myocardial infarction.

Similarly, each 50% increase in maximum turbidity was associated with increased risk of CV death/MI (HR 1.25; 95% CI 1.02–1.53) and CV death alone (HR 1.49; 95% CI 1.08–2.04). These relationships lost significance after adjustment for prognostic biomarkers. There was a numerical increase in bleeding in the lowest quartile group of maximum turbidity but this failed to reach statistical significance ( *p* = 0.15).

The prognostic value of fibrin clot lysis time was consistent regardless of randomised treatment, presentation, administration of LMWH within 2 days or invasive treatment (all interaction *p* > 0.1) ( Fig. 4 ).

**Fig. 5:**
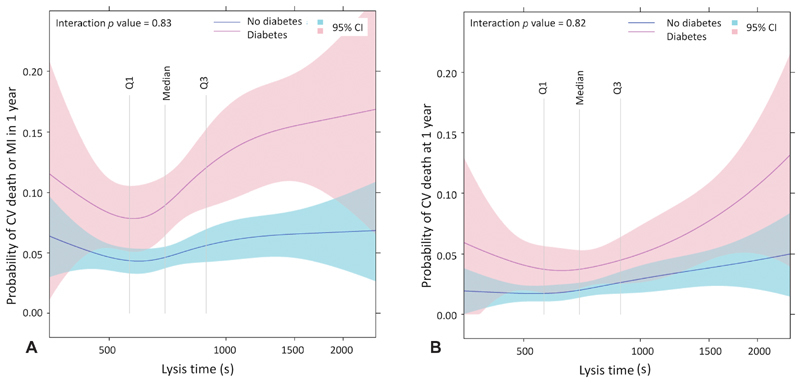
Relationship between fibrin clot lysis time and clinical outcomes according to diabetes status. One-year rates of cardiovascular (CV) death or spontaneous myocardial infarction (MI) ( **A** ) and CV death alone ( **B** ) in relation to lysis time, transformed using restricted cubic splines, according to diabetes status.

Similarly, the prognostic value of fibrin clot lysis time was consistent regardless of diabetes status but the magnitude of relationship appears to be higher in patients with diabetes ( Fig. 5 ).

### Fibrin Clot Properties over Time

Fibrin clot lysis time remained prolonged at 1 month (864 ± 406 seconds) compared with hospital discharge (863 ± 445 seconds) ( *p* = 0.64) despite a drop in overall maximum turbidity (0.44 ± 0.14 vs. 0.53 ± 0.2; *p* < 0.001).

## Discussion

For the first time, in a large cohort of ACS patients with diabetes, we have shown prolonged fibrin clot lysis to predict CV death and MI after adjustment for clinical risk predictors as well as traditional and novel prognostic biomarkers. We have also shown fibrin clot lysis time to remain prolonged at 1 month following ACS, despite a drop in fibrin clot turbidity. Being a functional assay, our methodology takes into account quantitative and qualitative changes in various clotting factors, thus giving an overall assessment of fibrin-related thrombosis risk. The independent association between impaired lysis and worse ischaemic outcomes may indicate that some patients may benefit from further optimisation of anti-thrombotic therapy, for example, by dropping dual anti-platelet therapy and replacing it with a combination of a single anti-platelet agent and an anticoagulant, since anticoagulant therapy potentiates fibrinolysis. 23 24 25 However, the safety and efficacy of such an approach requires further research.

Despite the large number of patients included in our study, the study lacks sufficient power to detect an independent relationship between individual components of the primary endpoint and fibrin clot properties. However, the trend of association with both CV death and MI is consistent.

Diabetes and prolonged lysis are both associated with many high-risk patient characteristics and prognostic biomarkers. We have adjusted for many potential confounders and although the association between prolonged fibrin clot lysis and CV death/spontaneous MI remained significant, including many confounders in adjustment models is a limitation. Reassuringly, the results are very similar with the many different models we tested for.

Another limitation of our methodology is that we studied fibrinolysis in plasma samples rather than whole blood. This may exclude the cellular effects on the fibrinolytic pathway. However, our results provide a ‘proof-of-concept’ of the importance of intrinsic fibrinolysis in predicting outcomes.

The very weak correlation between fibrin clot lysis potential and troponin levels indicates a possible pathophysiological link with worse outcomes that is independent of the size of MI. Fibrin clot maximum turbidity had a stronger relationship with CRP as well as troponin and NT-proBNP. This is to be expected as turbidity reflects fibrin clot density, which is more closely related to fibrinogen levels, and fibrinogen levels are well known to increase with inflammation, such as may be provoked by large MIs. 20

Although fibrin clot turbidity is only marginally higher in the diabetes population compared with individuals without diabetes, lysis time is significantly longer in the former group, consistent with previous studies. 7 26 There are several diabetes-specific mechanisms that lead to impaired clot lysis, including glycation of fibrinogen, which results in more compact clots that resist fibrinolysis, 27 increased incorporation of anti-fibrinolytic proteins into the clot 28 and glycation of plasminogen, which inhibits plasmin generation and modulates enzyme activity, thus further impairing the fibrinolytic process. 29 These previous findings may explain our results demonstrating a relationship between HbA1c and clot lysis time. Improving glycaemic control can modulate outcomes post-ACS and a reduction in fibrin clot lysis time may be one of the mechanisms involved. 30 Similarly, approaches to ameliorate inflammation may be successful at improving lysis potential. In studies involving human aortic endothelial cells, CRP resulted in increased plasminogen activator inhibitor-1 expression and reduction in tPA activity, which may explain the observed relationship between increased inflammation and pro-thrombotic changes in fibrin parameters. 31 32 Although diabetes was associated with increased maximum turbidity, there was no clear association between this fibrin parameter and glycaemic control. This suggests that the relationship between glycaemic control and lysis time is related to alteration in the fibrinolytic proteins rather than changes in fibrin clot structure in the cohort studied. However, it should be noted that over-treatment of high glucose levels and precipitation of hypoglycaemia can also impair fibrin clot lysis, 33 potentially explaining the inconsistent relationship between improved glycaemic control and outcome observed in clinical studies. 34 35 36

LDL cholesterol levels and platelet count increased with increasing lysis time. These observations are intriguing and support previously reported effects of cholesterol and increased platelet reactivity on fibrinolysis. 37 However, randomisation to ticagrelor was not associated with fibrinolysis potential and this suggests that platelet reactivity has limited impact on fibrin clot lysis in plasma. Statins and fenofibrates were previously shown to improve fibrinolysis. 38 We are unable to confirm this in our study as > 90% of patients were receiving statins but the relationship between increased lysis and increased LDL levels suggests a role for cholesterol-lowering therapy in modulating fibrinolysis.

Increased incorporation of anti-fibrinolytic proteins into the fibrin clot represents another mechanism for impaired fibrinolysis in diabetes. 39 Developing therapies against one or more of these proteins may represent a targeted approach that helps to improve prognosis in ACS patients with diabetes, while minimising bleeding risk.

The relationship between impaired fibrin clot lysis and female gender is consistent with previously observed results in other cohorts of high-risk vascular patients with type 2 diabetes. 26 40 However, another study, in a younger cohort, found no difference in lysis potential between males and females. 41 The latter study was much smaller than the other two, including a limited number of younger subjects who had type 1 rather than type 2 diabetes, and these differences in study populations are likely to explain the discrepancies. Prolonged lysis may be one mechanism for the reduction in CV protection in women with diabetes and further work is needed in this area to understand the exact pathways involved.

## Conclusion

Adverse fibrin clots that resist lysis predict CV death and MI in ACS patients with diabetes despite contemporary therapies. The relationship between high-risk vascular conditions and impaired lysis provide potential mechanistic insights into recurrent events. The weak correlation between fibrin clot lysis potential and troponin indicates that the association with worse outcomes is relatively independent of the magnitude of MI. Developing strategies to improve lysis tendency may help improve prognosis in high-risk ACS patients and future research in this area is warranted.

## Notes

**What is known about this topic?**

- Diabetes is associated with worse outcomes following ACS.
- The relationship between fibrin clot properties and clinical outcomes in diabetes patients presenting with ACS remains unknown.

**What does this paper add?**

- Prolonged fibrin clot lysis predicts cardiovascular death and spontaneous myocardial infarction in diabetes patients presenting with ACS.
- Further research is needed to establish the suitability of targeting reduced fibrinolysis to aid improve outcomes in diabetes patients with ACS.

## References

[1] Jernberg T Hasvold P Henriksson M Hjelm H Thuresson M Janzon M Cardiovascular risk in post-myocardial infarction patients: nationwide real world data demonstrate the importance of a long-term perspective Eur Heart J 2015 36 19 1163 1170 25586123 10.1093/eurheartj/ehu505

[2] Sprafka J M Burke G L Folsom A R McGovern P G Hahn L P Trends in prevalence of diabetes mellitus in patients with myocardial infarction and effect of diabetes on survival. The Minnesota Heart Survey Diabetes Care 1991 14 07 537 543 1914792 10.2337/diacare.14.7.537

[3] McGuire D K Emanuelsson H Granger C B Influence of diabetes mellitus on clinical outcomes across the spectrum of acute coronary syndromes. Findings from the GUSTO-IIb study. GUSTO IIb Investigators Eur Heart J 2000 21 21 1750 1758 11052839 10.1053/euhj.2000.2317

[4] James S Angiolillo D J Cornel J H Ticagrelor vs. clopidogrel in patients with acute coronary syndromes and diabetes: a substudy from the PLATelet inhibition and patient Outcomes (PLATO) trial Eur Heart J 2010 31 24 3006 3016 20802246 10.1093/eurheartj/ehq325 PMC3001588

[5] Colwell J A Halushka P V Sarji K Levine J Sagel J Nair R M Altered platelet function in diabetes mellitus Diabetes 1976 25(2 Suppl):826 831 823064

[6] Sagel J Colwell J A Crook L Laimins M Increased platelet aggregation in early diabetes mellitus Ann Intern Med 1975 82 06 733 738 1138583 10.7326/0003-4819-82-6-733

[7] Alzahrani S H Ajjan R A Coagulation and fibrinolysis in diabetes Diab Vasc Dis Res 2010 7 04 260 273 20847109 10.1177/1479164110383723

[8] Ibanez B James S Agewall S 2017 ESC Guidelines for the management of acute myocardial infarction in patients presenting with ST-segment elevation: the Task Force for the management of acute myocardial infarction in patients presenting with ST-segment elevation of the European Society of Cardiology (ESC) Eur Heart J 2018 39 02 119 177 28886621 10.1093/eurheartj/ehx393

[9] Valgimigli M Bueno H Byrne R A 2017 ESC focused update on dual antiplatelet therapy in coronary artery disease developed in collaboration with EACTS: the Task Force for dual antiplatelet therapy in coronary artery disease of the European Society of Cardiology (ESC) and of the European Association for Cardio-Thoracic Surgery (EACTS) Eur Heart J 2018 39 03 213 260 28886622 10.1093/eurheartj/ehx419

[10] Subherwal S Bach R G Chen A Y Baseline risk of major bleeding in non-ST-segment-elevation myocardial infarction: the CRUSADE (Can Rapid risk stratification of Unstable angina patients Suppress ADverse outcomes with Early implementation of the ACC/AHA Guidelines) Bleeding Score Circulation 2009 119 14 1873 1882 19332461 10.1161/CIRCULATIONAHA.108.828541 PMC3767035

[11] Wiviott S D Braunwald E Angiolillo D J Greater clinical benefit of more intensive oral antiplatelet therapy with prasugrel in patients with diabetes mellitus in the trial to assess improvement in therapeutic outcomes by optimizing platelet inhibition with prasugrel-Thrombolysis in Myocardial Infarction 38 Circulation 2008 118 16 1626 1636 18757948 10.1161/CIRCULATIONAHA.108.791061

[12] Mega J L Braunwald E Wiviott S D Rivaroxaban in patients with a recent acute coronary syndrome N Engl J Med 2012 366 01 9 19 22077192 10.1056/NEJMoa1112277

[13] Wallentin L Lindholm D Siegbahn A Biomarkers in relation to the effects of ticagrelor in comparison with clopidogrel in non-ST-elevation acute coronary syndrome patients managed with or without in-hospital revascularization: a substudy from the Prospective Randomized Platelet Inhibition and Patient Outcomes (PLATO) trial Circulation 2014 129 03 293 303 24170388 10.1161/CIRCULATIONAHA.113.004420

[14] Collet J P Allali Y Lesty C Altered fibrin architecture is associated with hypofibrinolysis and premature coronary atherothrombosis Arterioscler Thromb Vasc Biol 2006 26 11 2567 2573 16917107 10.1161/01.ATV.0000241589.52950.4c

[15] Fatah K Silveira A Tornvall P Karpe F Blombäck M Hamsten A Proneness to formation of tight and rigid fibrin gel structures in men with myocardial infarction at a young age Thromb Haemost 1996 76 04 535 540 8902992

[16] Undas A Plicner D Stepień E Drwiła R Sadowski J Altered fibrin clot structure in patients with advanced coronary artery disease: a role of C-reactive protein, lipoprotein(a) and homocysteine J Thromb Haemost 2007 5 09 1988 1990 17723142 10.1111/j.1538-7836.2007.02637.x

[17] Neergaard-Petersen S Ajjan R Hvas A M Fibrin clot structure and platelet aggregation in patients with aspirin treatment failure PLoS One 2013 8 08 e71150 23976993 10.1371/journal.pone.0071150 PMC3747207

[18] Leander K Blombäck M Wallén H He S Impaired fibrinolytic capacity and increased fibrin formation associate with myocardial infarction Thromb Haemost 2012 107 06 1092 1099 22476576 10.1160/TH11-11-0760

[19] Undas A Zalewski J Krochin M Altered plasma fibrin clot properties are associated with in-stent thrombosis Arterioscler Thromb Vasc Biol 2010 30 02 276 282 19910643 10.1161/ATVBAHA.109.194936

[20] Sumaya W Wallentin L James S K Fibrin clot properties independently predict adverse clinical outcome following acute coronary syndrome: a PLATO substudy Eur Heart J 2018 39 13 1078 1085 29390064 10.1093/eurheartj/ehy013 PMC6019045

[21] Wallentin L Becker R C Budaj A Ticagrelor versus clopidogrel in patients with acute coronary syndromes N Engl J Med 2009 361 11 1045 1057 19717846 10.1056/NEJMoa0904327

[22] James S Akerblom A Cannon C P Comparison of ticagrelor, the first reversible oral P2Y(12) receptor antagonist, with clopidogrel in patients with acute coronary syndromes: rationale, design, and baseline characteristics of the PLATelet inhibition and patient Outcomes (PLATO) trial Am Heart J 2009 157 04 599 605 19332184 10.1016/j.ahj.2009.01.003

[23] Sumaya W Parker W AE Fretwell R Pharmacodynamic effects of a 6-hour regimen of enoxaparin in patients undergoing primary percutaneous coronary intervention (PENNY PCI Study) Thromb Haemost 2018 118 07 1250 1256 29874689 10.1055/s-0038-1657768 PMC6202933

[24] Varin R Mirshahi S Mirshahi P Clot structure modification by fondaparinux and consequence on fibrinolysis: a new mechanism of antithrombotic activity Thromb Haemost 2007 97 01 27 31 17200767

[25] Varin R Mirshahi S Mirshahi P Whole blood clots are more resistant to lysis than plasma clots--greater efficacy of rivaroxaban Thromb Res 2013 131 03 e100 e109 23313382 10.1016/j.thromres.2012.11.029

[26] Neergaard-Petersen S Hvas A M Kristensen S D The influence of type 2 diabetes on fibrin clot properties in patients with coronary artery disease Thromb Haemost 2014 112 06 1142 1150 25187394 10.1160/TH14-05-0468

[27] Pieters M van Zyl D G Rheeder P Glycation of fibrinogen in uncontrolled diabetic patients and the effects of glycaemic control on fibrinogen glycation Thromb Res 2007 120 03 439 446 17156827 10.1016/j.thromres.2006.10.016

[28] Hess K Alzahrani S H Mathai M A novel mechanism for hypofibrinolysis in diabetes: the role of complement C3 Diabetologia 2012 55 04 1103 1113 21918806 10.1007/s00125-011-2301-7

[29] Dunn E J Philippou H Ariëns R A Grant P J Molecular mechanisms involved in the resistance of fibrin to clot lysis by plasmin in subjects with type 2 diabetes mellitus Diabetologia 2006 49 05 1071 1080 16538489 10.1007/s00125-006-0197-4

[30] Bouida W Beltaief K Msolli M A One-year outcome of intensive insulin therapy combined to glucose-insulin-potassium in acute coronary syndrome: a randomized controlled study J Am Heart Assoc 2017 6 11 6 10.1161/JAHA.117.006674 PMC5721763 29138181

[31] Devaraj S Xu D Y Jialal I C-reactive protein increases plasminogen activator inhibitor-1 expression and activity in human aortic endothelial cells: implications for the metabolic syndrome and atherothrombosis Circulation 2003 107 03 398 404 12551862 10.1161/01.cir.0000052617.91920.fd

[32] Singh U Devaraj S Jialal I C-reactive protein decreases tissue plasminogen activator activity in human aortic endothelial cells: evidence that C-reactive protein is a procoagulant Arterioscler Thromb Vasc Biol 2005 25 10 2216 2221 16123325 10.1161/01.ATV.0000183718.62409.ea

[33] Chow E Iqbal A Walkinshaw E Prolonged prothrombotic effects of antecedent hypoglycemia in individuals with type 2 diabetes Diabetes Care 2018 41 12 2625 2633 30327358 10.2337/dc18-0050

[34] Malmberg K Rydén L Efendic S Randomized trial of insulin-glucose infusion followed by subcutaneous insulin treatment in diabetic patients with acute myocardial infarction (DIGAMI study): effects on mortality at 1 year J Am Coll Cardiol 1995 26 01 57 65 7797776 10.1016/0735-1097(95)00126-k

[35] Malmberg K Rydén L Wedel H Intense metabolic control by means of insulin in patients with diabetes mellitus and acute myocardial infarction (DIGAMI 2): effects on mortality and morbidity Eur Heart J 2005 26 07 650 661 15728645 10.1093/eurheartj/ehi199

[36] Patel A MacMahon S Chalmers J Intensive blood glucose control and vascular outcomes in patients with type 2 diabetes N Engl J Med 2008 358 24 2560 2572 18539916 10.1056/NEJMoa0802987

[37] Panes O Padilla O Matus V Clot lysis time in platelet-rich plasma: method assessment, comparison with assays in platelet-free and platelet-poor plasmas, and response to tranexamic acid Platelets 2012 23 01 36 44 21787173 10.3109/09537104.2011.596957

[38] Undas A Celinska-Löwenhoff M Löwenhoff T Szczeklik A Statins, fenofibrate, and quinapril increase clot permeability and enhance fibrinolysis in patients with coronary artery disease J Thromb Haemost 2006 4 05 1029 1036 16689755 10.1111/j.1538-7836.2006.01882.x

[39] Kearney K Tomlinson D Smith K Ajjan R Hypofibrinolysis in diabetes: a therapeutic target for the reduction of cardiovascular risk Cardiovasc Diabetol 2017 16 01 34 28279217 10.1186/s12933-017-0515-9 PMC5345237

[40] Alzahrani S H Hess K Price J F Gender-specific alterations in fibrin structure function in type 2 diabetes: associations with cardiometabolic and vascular markers J Clin Endocrinol Metab 2012 97 12 E2282 E2287 22996148 10.1210/jc.2012-2128

[41] Tehrani S Jörneskog G Ågren A Lins P E Wallén H Antovic A Fibrin clot properties and haemostatic function in men and women with type 1 diabetes Thromb Haemost 2015 113 02 312 318 25318636 10.1160/TH14-05-0404

